# Telephone follow-up to a mail survey: when to offer an interview compared to a reminder call

**DOI:** 10.1186/1471-2288-12-32

**Published:** 2012-03-20

**Authors:** Jeanette Y Ziegenfuss, Kelly R Burmeister, Ann Harris, Stefan D Holubar, Timothy J Beebe

**Affiliations:** 1 Division of Health Care Policy & Research, Mayo Clinic, 200 First Street SW, Rochester, MN, 55904, USA; 2 Children's Hospital Boston, 300 Longwood Ave, Boston, MA, 02115, USA; 3 Department of General Surgery, Dartmouth-Hitchcock Medical Center, One Medical Center Drive, Lebanon, NH, 03756, USA

**Keywords:** Survey methods, Telephone follow-up, Health surveys, Survey Nonresponse follow-up

## Abstract

**Background:**

Using a different mode of contact on the final follow-up to survey non-respondents is an identified strategy to increase response rates. This study was designed to determine if a reminder phone call or a phone interview as a final mode of contact to a mailed survey works better to increase response rates and which strategy is more cost effective.

**Methods:**

A randomized study was embedded within a survey study of individuals treated with ulcerative colitis conducted in March 2009 in Olmsted County, Minnesota. After two mail contacts, non-respondents were randomly assigned to either a reminder telephone call or a telephone interview. Average cost per completed interview and response rates were compared between the two experimental conditions.

**Results:**

The response rate in the reminder group and the interview did not differ where we considered both a completed survey and a signed form a complete (24% vs. 29%, p = 0.08). However, if such a signed form was not required, there was a substantial advantage to completing the interview over the phone (24% vs. 43%, p < 0.0001). The reminder group on average cost $27.00 per completed survey, while the interview group on average cost $53.00 per completed survey when a signed form was required and $36.00 per complete when a signed form was not required.

**Conclusions:**

The additional cost of completing an interview is worth it when an additional signed form is not required of the respondent. However, when such a signed form is required, offering an interview instead of a reminder phone call as a follow up to non-respondents does not increase response rates enough to outweigh the additional costs.

## Background

Although response rates vary by study and mode of administration, there is evidence that response rates are declining across all modes of survey administration [1-3]. Mixed mode surveys have been shown to increase response rates by appealing to respondents who did not respond to the first mode they were offered [4]. Thus switching modes can offer potential respondents an additional opportunity for survey completion and allow respondents to complete the survey utilizing a mode with which they may feel more comfortable.

Dillman and colleagues [5] suggest making the final contact to a potential respondent distinct from previous failed attempts as a way to increase response rates based on the premise that people prefer different modes of data collection. While historically in mail surveys this took the form of final contact by certified mail or more recently delivery service, they recommend using a telephone contact when telephone numbers are available [5]. In a recent meta-analysis, telephone reminders were found to have no overall impact on response rate compared to no reminder [6]. However, the results are somewhat inconclusive as they are only based on three studies and the findings are in opposing directions [6]. Although generally more expensive than additional mail contacts on a per unit basis, telephone reminders can result in reduced costs when considering cost per completed survey. One study found a telephone reminder was associated with a significant increase in response rates and with a marginal cost savings of 15% lower costs per completed survey compared to when no reminder phone call was used [7].

Conventional wisdom in the survey research field dictates that once a potential respondent is on the phone for a reminder, completing an interview over the phone is an attractive option as not doing so may present a missed opportunity. In fact, Dillman and colleagues further suggest completing an interview over the telephone as a mechanism to increase response rates, albeit with increased implementation and completion costs due to the use of interviewers to administer the survey versus merely reminding prospective respondents to fill out the mailed form [5]. In this study, using a randomized design, we compare the relative effectiveness and cost per complete survey of phone reminders compared to phone interviews at the third contact of a mailed survey.

## Methods

The present methodological study was embedded within a larger study conducted in March 2009 by Holubar and colleagues assessing the influence of referral bias on patient reported outcomes among patients treated for ulcerative colitis [8]. The survey was conducted in Olmsted County, Minnesota based on a frame established using the resources of the Rochester Epidemiology Project (REP), the medical record system for health care providers to residents of Olmsted County [9]. The REP was used to identify study patients who were either a resident of Olmsted County or referral-center patients who underwent a surgical procedure (ileal pouch-anal anastomosis or IPAA) in the past 20 years. Respondents were selected from the REP (N = 1825) and after two mailings, 873 non-responders were randomized to one of two conditions: a telephone reminder (N = 438) or a telephone interview (N = 435). This study was approved by the Mayo Clinic and Olmsted Clinic Institutional Review Boards.

### Analytical strategy

In the larger study, only surveys with both a signed Health Insurance Portability and Accountability Act (HIPAA) authorization form (HAF) and a completed interview or survey were considered complete. For the purposes of this analysis and to make the results generalizable to institutions that do not require a signed form, we consider two definitions of what it means to be a respondent: 1) *generalizable respondent: *completed survey or interview regardless of HAF status, and 2) *study respondent: *completed survey or interview *and *signed form returned. While a survey could be completed only in the paper mode within the reminder group, it could be completed either as a telephone interview or by paper within the interview group if a respondent chose to not complete the phone interview even if they were randomized to that arm. Response rates and costs were compared between reminder and interview groups under each definition of respondent (generalizable and study respondent). Marginal cost per completed survey was calculated by multiplying the cost per hour of telephone interviewers (who also completed the reminder phone calls) by the total number of hours worked by the interviewers in each condition.

The response rates for both the generalizable and study respondent definitions were compared between the two conditions overall and within age and gender subpopulations using Chi-square tests of independence. All analyses were conducted using SAS 9.1 statistical software. Unadjusted p-values are reported. Within each set of analyses, 14 total statistical tests were performed. P-values less than 0.0036 (= 0.05/14) were considered statistically significant using a Bonferroni adjustment for multiple testing. Raw p-values less than 0.05 were also noted as indicating potentially important differences. All variables were treated as categorical including age, gender, and return of signed form.

## Results

Overall 9% of individuals refused the survey. An additional 30% could not be contacted or were lost to follow-up. These outcomes did not differ across the interview and reminder groups. The survey response rate (Table 1) in the reminder group and the interview group were not significantly different using our definition of a study respondent where we received both a completed survey and a signed form (24% vs. 29%, p = 0.08). The reminder group on average cost $27.00 per completed survey, while the interview group on average cost $53.00 per completed survey. In contrast, there was a significant difference in response rate when using the generalizable definition of a respondent that included a survey response regardless of receipt or non-receipt of a signed form (24% vs. 43%, p < 0.0001). The cost per complete in the reminder group was still $27.00 while the cost per complete in the interview group was reduced to $36.00.

**Table 1:** Response rate by respondent population and demographic subpopulations

	Interview Group (n = 435)		Reminder Group (n = 438)		
	**n (%) response**	p*	**n (%) response**	p*	p**

STUDY RESPONDENT: Completed Survey and HIPAA Authorization Form					

Total	128 (29.4%)		106 (24.2%)		0.08

Mail Survey	23 (5.3%)		106 (24.2%)		< 0.0001

Phone Interview	105 (24.1%)		NA		(only applies to interview group)

Gender					

Male	65 (26.2%)	0.09	65 (23.5%)	0.64	0.47
				
Female	63 (33.7%)		41 (25.5%)		0.10

Age Group					

18-24	6 (31.6%)	0.03	4 (36.4%)	0.01	0.79
		
25-34	18 (26.1%)		22 (29.0%)		0.70
		
35-44	23 (20.4%)		15 (11.8%)		0.07
		
45-54	37 (28.9%)		34 (28.3%)		0.92
		
55-64	32 (42.1%)		22 (31.4%)		0.18
		
65+	12 (40.0%)		9 (26.5%)		0.25

GENERALIZABLE RESPONDENT: Completed Survey Regardless of HIPAA Authorization Form					

Total	187 (43.0%)		106 (24.2%)		< 0.0001

Mail Survey	24 (5.5%)		106 (24.2%)		0.50

Phone Interview	163 (37.5%)		NA		(only applies to interview group)

Gender					

Male	97 (39.1%)	0.06	65 (23.5%)	0.64	< 0.001

Female	90 (48.1%)		41 (25.5%)		< 0.001

Age Group					

18-24	9 (47.4%)	0.08	4 (36.4%)	0.01	0.56

25-34	28 (40.6%)		22 (29.0%)		0.14

35-44	39 (34.5%)		15 (11.8%)		< 0.001

45-54	53 (41.4%)		34 (28.3%)		0.03

55-64	42 (55.3%)		22 (31.4%)		0.004

65+	16 (53.3%)		9 (26.5%)		0.03

* Within group

** Between groups

Using either definition of respondent, response rates for completed surveys were not significantly different within reminder and interview group respectively for either age or gender (Table 1). There were no between group differences using the study definition of responder. There were, however higher response rates for some subpopulations using the generalizable definition of respondent. Specifically, the response rate was higher in the interview group for both males and females as well as those individuals 35-44 and 55-64. (Table 1).

## Discussion

The response rates were five percentage points higher for the interview group than the reminder group using the conservative definition of a respondent (i.e., received both a completed survey and a signed form; however this difference did not reach statistical significance. Thus, conclusions regarding whether the response rate was higher due to chance or due to the option of completing the survey in an alternate mode (i.e. telephone) remains uncertain. What we did observe was a two-fold difference in the cost per completion in the interview versus reminder condition. Specifically, the interview group cost on average $53.00 per completed interview compared to an average of $27.00 per completed interview in the reminder group. Applying this difference to the entire sample, had we deployed an interviewing strategy in comparison to a reminder phone call as a matter of course, our total study cost would have been $7,844 (137%) more expensive. This could represent a financial challenge to investigators and studies constrained by limited resources.

In contrast, had we not been constrained to using only those surveys for which we also received a signed form we would have obtained a 19 percentage point increase in response rate in the interview group compared to the reminder group. This translated into only an additional $9.00 per complete in the interview group (for a total of $36.00 per complete). Thus, the decision to complete a telephone interview instead of just a reminder phone call hinges on whether or not prospective respondents are required to mail in a signed consent or authorization form along with the completed survey (the former distinguishes our study definition of a respondent from the generalizable definition).

We saw that in the case where a signed form was required, reminder telephone calls, while not negatively impacting response rates, were a more cost-effective way to achieve survey completion than offering a telephone interview. Moreover, reminder phone calls do not change the mode (i.e. self-administered via mail used in the first two contacts) of survey completion while interviews do. Therefore potential response pattern differences due to mode are nonexistent when using reminder calls, an additional benefit to reminders versus interviews. As there is evidence that response patterns can differ when an interviewer is present compared to a self-administered survey [4], implementing mixed mode data collection through a final telephone interview increases the risks of different response patterns across contact waves.

## Conclusions

Although the strength and generalizability of our findings may be limited by the very specialized nature of our sample (patients who underwent ileal pouch-anal anastomosis), our study represents one of the first formal investigations assessing the relative merits of interviewing versus reminder call in bringing about enhanced participation. We conclude that offering a telephone interview instead of a reminder phone call as a follow-up to survey non-response did not increase response rates enough to outweigh the additional costs when a signed form is required. However, in instances where such a mail form is not required, it behooves researchers to complete an interview with a respondent when they have them on the phone.

## Abbreviations

REP: Rochester Epidemiology Project; HIPAA: Health Insurance Portability and Accountability Act; HAF: HIPAA authorization form.

## Competing interests

The authors declare that they have no competing interests.

## Authors' contributions

AH conceptualized study design with assistance from TJB and SDH. TJB and AH oversaw study implementation. SDH led the parent study in which this study was embedded. JYZ directed analysis and interpretation of findings. KRB, AH and TJB participated in interpretation of findings. JYZ wrote the manuscript with assistance from KRB and TJB. All authors read and approved the final manuscript.

## Pre-publication history

The pre-publication history for this paper can be accessed here:

http://www.biomedcentral.com/1471-2288/12/32/prepub
